# Influence of pH Adjustment Parameter for Sol–Gel Modification on Structural, Microstructure, and Magnetic Properties of Nanocrystalline Strontium Ferrite

**DOI:** 10.1186/s11671-018-2562-x

**Published:** 2018-05-23

**Authors:** Raba’ah Syahidah Azis, Sakinah Sulaiman, Idza Riati Ibrahim, Azmi Zakaria, Jumiah Hassan, Nor Nadhirah Che Muda, Rodziah Nazlan, Norlaily M. Saiden, Yap Wing Fen, Muhammad Syazwan Mustaffa, Khamirul Amin Matori

**Affiliations:** 1 Institute of Advanced Technology, Universiti Putra Malaysia, 43400 UPM Serdang, Selangor Malaysia; 2 Departments of Physics, Faculty of Science, Universiti Putra Malaysia, 43400 UPM Serdang, Selangor Malaysia; 3 Faculty of Industrial Sciences & Technology, Universiti Malaysia Pahang, Lebuhraya Tun Razak, Gambang, 26300 Kuantan, Malaysia

**Keywords:** Sol–gel, pH, Structural, Microstructure, Magnetic behavior, Strontium hexaferrite (SrFe_12_O_19_)

## Abstract

Synthesis of nanocrystalline strontium ferrite (SrFe_12_O_19_) via sol–gel is sensitive to its modification parameters. Therefore, in this study, an attempt of regulating the pH as a sol–gel modification parameter during preparation of SrFe_12_O_19_ nanoparticles sintered at a low sintering temperature of 900 °C has been presented. The relationship of varying pH (pH 0 to 8) on structural, microstructures, and magnetic behaviors of SrFe_12_O_19_ nanoparticles were characterized by X-ray diffraction (XRD), field emission scanning microscope (FESEM), and vibrating sample magnetometer (VSM). Varying the pH of precursor exhibited a strong effect on the sintered density, crystal structure and magnetic properties of the SrFe_12_O_19_ nanoparticles. As the pH is 0, the SrFe_12_O_19_ produced relatively largest density, saturation magnetization, *M*_s_, and coercivity, *H*_c_, at a low sintering temperature of 900 °C. The grain size of SrFe_12_O_19_ is obtained in the range of 73.6 to 133.3 nm. The porosity of the sample affected the density and the magnetic properties of the SrFe_12_O_19_ ferrite. It is suggested that the low-temperature sintered SrFe_12_O_19_ at pH 0 displayed *M*_s_ of 44.19 emu/g and *H*_c_ of 6403.6 Oe, possessing a significant potential for applying in low-temperature co-fired ceramic permanent magnet.

## Highlight

- Synthesis of strontium ferrite (SrFe_12_O_19_) nanoparticles using sol–gel auto combustion technique.
- The SrFe_12_O_19_ nanoferrite phase was obtained at a low sintering temperature, 900 °C.
- Magnetic parameter of saturation magnetization *M*_s_, remnant *M*_r_, and coercivity *H*_c_ decrease as pH increases.

## Background

Strontium ferrite (SrFe_12_O_19_) has been extensively studied for their potential applications in microwave devices, high-density magnetic recording, electronic devices, and permanent magnet. Permanent magnet ferrites are widely used in the electrical manufacturing industry due to its several advantages [1] and impressive properties such as high electrical resistivity [2], large hysteresis loss, and high intrinsic coercivity [3]. It is best known as a good heat resistance and corrosion resistance and useful for many applications. Strontium ferrite has attracted more scientific studies in recent years due to its high magnetic anisotropy, which is responsible for the high coercivity of crystalline structure [4, 5] and thus can ensure a high coercivity even when the size of the particles is reduced into nanoscale with single-domain structure. The ferromagnetism exhibited by SrFe_12_O_19_ is attributed to the Fe^3+^ ion sublattices present in the structure. They are distributed in three octahedral (12 k, 2a, 4f_2_), one tetrahedral (4f_1_), and one bipyramidal sites (2b). From these sites, 12 k, 2a, and 2b are represented as the high spin states and 4f_1_ and 4f_2_ are considered as the low spin states [6, 7]. The magnetic moments of the Fe^3+^ ions are coupled to each other by super-exchange interactions mediated by O^2−^ ions. The Sr^2+^ ion is responsible for the large magnetic uniaxial anisotropy as it causes a perturbation of the crystal lattice [8]. Strontium hexaferrite (SrFe_12_O_19_) nanoparticles have a mean particle size of less than 0.1 μm and are made of homogeneous particle size distribution [9]. The smaller particle size produces a large surface area, significantly enhancing the SrFe_12_O_19_ nanoparticles properties, such as its chemical, physical, mechanical, and magnetic properties, resulting in interesting properties for nanoferrite applications.

The conventional ceramic solid-state method is difficult in obtaining nanoparticles and mono-sized particles [4, 5]. It has limitations such as long heating schedule at high sintering temperature of about 1300 °C, higher obtained grain/particle size, and higher time consumption. The experimental conditions involved in the making of the ferrite nanoparticles play a key role in the resulting properties as well as the particle size of the ferrite nanoparticles. In order to achieve highly homogeneous SrFe_12_O_19_ nanoparticles consisting of a single-domain structure at low sintering or calcination temperature, various methods have been introduced so that a wide grain size distribution with anomolous grain growth promoted during sintering could be avoided. The methods include co-precipitation [9, 10], salt-melt method [11], hydrothermal [12, 13], microemulsion [14], and sol–gel process [1, 4, 15]. Among these methods, sol–gel route is a low-cost, simple, and reliable method to control the stoichiometry and to produce nanocrystalline ferrite. The sol–gel process produces a homogeneous mixed oxide that can lower the calcination temperature and produce a smaller crystallite size [3]. Optimizing the molar ratio of Fe to Sr (Fe/Sr) is very important to produce a single-phase sample, ultrafine particle, and lower calcination temperatures [1]. This ratio varies with change in starting materials and with change in method of production [1]. At high calcination temperature, both grain size and exchange coupling increase. These will be unfavorable for getting a good quality of permanent magnet [16]. In general, metal alkoxides are often used as raw materials in sol–gel process, but many of the alkoxides are very difficult to be obtained and dealt with because of the high sensitivity to the atmospheric moisture. Moreover, it is not easy to control the rate of alkoxide hydrolysis when multi-component ceramics are to be prepared. Metal salts are employed in this study since they are very useful, cheaper, and easier to handle. Besides, metal salts can be dissolved in many kinds of organic solvents, thus forming metal complexes by chelating the metal ions with organic ligands [17]. There are several sol–gel modification processes have been reported, such as pH adjustment [1, 18], basic agent [3], surfactant [1], carboxylic acid [2], and starting metal salts [3], to reduce the final calcination temperature, crystallite size [2], and high anisotropy of SrFe_12_O_19_ nanoparticles [12]. In sol–gel methods, the ability to form hydroxides and/or oxides strongly depends on the pH of the solution and the charge/radius ratio of the metal cation [17]. Furthermore, the pH of sol controls the amount of H^+^ or OH^−^ ions in the sol that effectively determines the polymerization of the metal–oxygen bonds. Also, it is known that during the sol–gel process, complexing process with citric acid is sensitive to pH values [19, 20]. Therefore, homogeneity of the sol which is essential for phase formation would be determined by the pH of the solution. It is well known that the magnetic properties of SrFe_12_O_19_ are strongly dependent on its morphology, particle/grain size, shape, orientation, and domain configurations by modifying the synthesis parameters. Therefore, in this work, we intend to regulate the pH of the solution as a sol–gel modification parameter to produce nanocrystalline ferrite with considerable values of magnetic properties at a lower calcination temperature.

## Methods

The experimental sequences of this study consisted of two major stages which were the synthesis of strontium ferrite nanoparticles via the sol–gel method (the “Synthesis of Strontium Ferrite Nanoparticles” section) and were followed by the characterizations of structural, microstructure, and magnetic properties of prepared strontium ferrite (the “Characterizations of Strontium Ferrite” section).

### Synthesis of Strontium Ferrite Nanoparticles

**Fig. 1:**
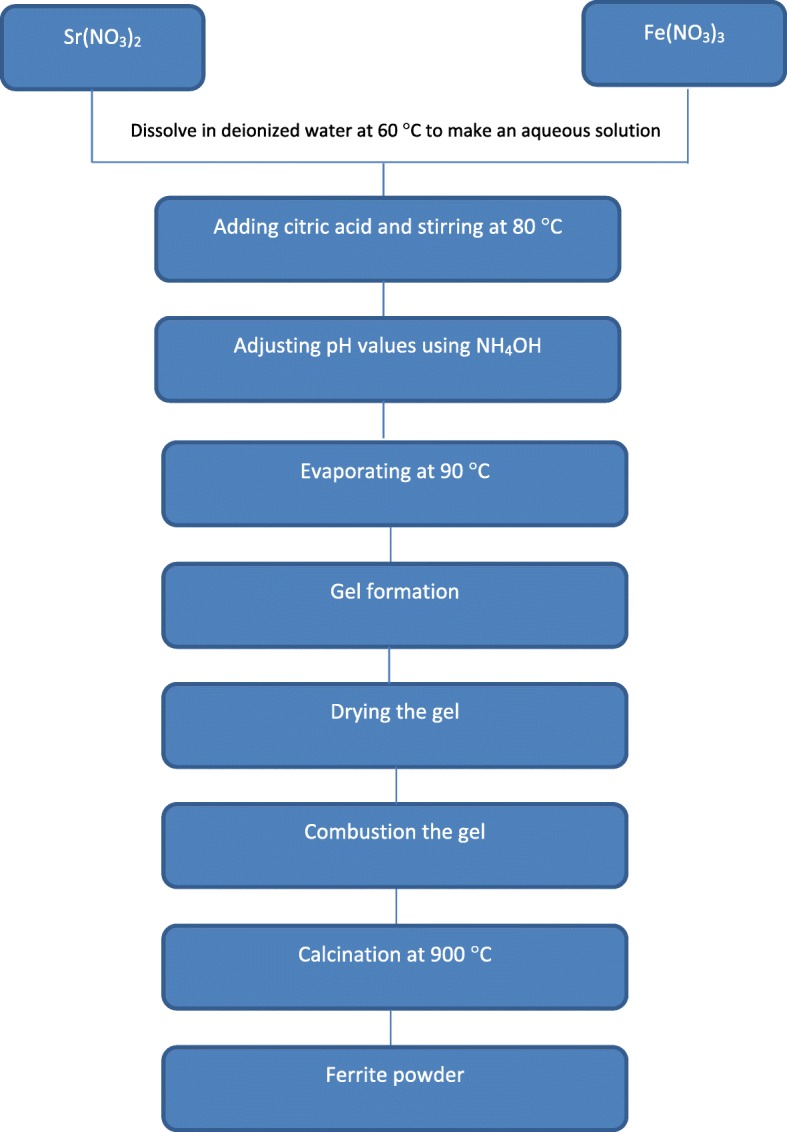
Flowchart for preparing SrFe_12_O_19_ nanoparticles powders by sol–gel method

Strontium ferrite nanoparticles have been synthesized via the sol–gel method. In this method, strontium nitrate anhydrous granular Sr(NO_3_)_2_ (98%, Alfa Aeser), iron(III) nitrate Fe(NO_3_)_3_ (99%, HmbG), citric acid C_3_H_4_(OH)(COOH)_3_ (99%, Alfa Aeser), ammonia NH_4_OH (25%, SYSTERM), and deionized water were used as starting materials for the preparation of the sample. Appropriate amounts of Sr(NO_3_)_2_ and Fe(NO_3_)_3_ are dissolved in 100 ml of deionized water for a few minutes at 60 °C with a constant stirrer rotation of 250 rpm to make an aqueous solution. Citric acid was added as a chelating agent with molar ratio of citrate to nitrate (C/N = 0.75), and the temperature was raised to 80 °C. The mixtures were continuously stirred, and NH_4_OH was added in order to vary the pH from pH 0 to pH 8. The pH was measured by HI2211 pH/ORP meter (HANNA instruments). The solutions were continuously stirred and heated for several hours at 90 °C, and the solution slowly turned into green sticky gel. Upon the formation of a dense sticky gel, the temperature of the hot plate is then increased up to 200 °C and combusted the gels for an hour for the dehydration process. The obtained powders were calcined at 900 °C for 6 h with the heating rate of 5 °C/min. A step-wise description of the synthesis procedure of SrFe_12_O_19_ nanoparticles is shown in Fig. 1.

### Characterizations of Strontium Ferrite

Characterization measurement of strontium ferrite has been carried out in terms of its structural, microstructure, and magnetic properties. The detailed explanation is shown in the following subsections.

#### Structural Properties

The structural characterization of the samples was characterized by X-ray diffraction (XRD) technique using a Philips X’pert X-Ray Diffractometer Model 7602 EA Almelo with Cu Kα radiation at 1.5418 Å. The range of diffraction angle used is from 20° to 80° at room temperature. The accelerating current and working voltage were 35 mA and 4.0 kV respectively. The data were analyzed by using an X’Pert High Score Plus software.

Fourier transform infrared (FTIR) characterization was carried out by a Perkin Elmer Fourier transform infrared spectrometer model 1650 to determine the infrared spectrum of absorption and emission bands of the sample. It was performed between infrared spectra of 280–4000 cm^−1^.

#### Microstructure Properties

The microstructural observation was performed by a field emission scanning electron microscope (FESEM) using a FEI Nova NanoSEM 230 machine. The distribution of grain size image was fixed at a magnification of 100kx with 5.0 kV accelerating voltage. The distributions of grain sizes were obtained by taking 200 different grain images for the sample and estimating the mean diameters of individual grains by using the imageJ software. The grain size distribution was measured by a mean linear intercept method.

#### Density

The density was measured using a Hildebrand Densitometer Model H-300 S. The density of the sintered pellet was obtained using the Archimedes principle with water as the fluid medium by Eq. 1,

1 $$ {\rho}_{\mathrm{exp}}=\left(\frac{W_{\mathrm{air}}-{W}_{\mathrm{water}}}{W_{\mathrm{water}}}\right)\times {\rho}_w $$

*ρ*_exp_ is the measured sample’s density, *ρ*_*w*_ is the density of water, *W*_air_ is the sample’s weight in air, and *W*_water_ is the sample’s weight in water.

#### Magnetic Properties

The magnetic properties of samples were measured by a vibrating sample magnetometer (VSM) Model 7404 LakeShore. The measurement was carried out at room temperature. The 12 kOe external field was applied parallel to the sample.

## Results and Discussion

### Structural Analysis

**Fig. 2:**
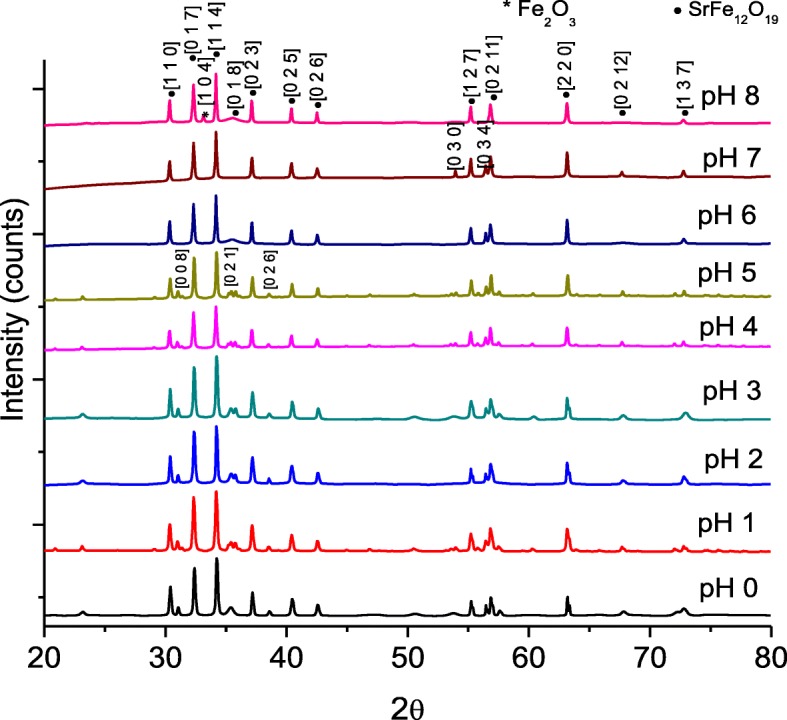
The X-ray diffraction spectra of SrFe_12_O_19_ for pH 0 to pH 8, sintered at 900 °C

Figure 2 shows the X-ray diffraction (XRD) spectra of SrFe_12_O_19_ nanocrystalline by varying the pH. The structure of XRD peaks was referred to standard SrFe_12_O_19_ with JCPDS reference code of 98-004-3603. The characteristic peaks and miller indices of SrFe_12_O_19_ are also shown in the figure. The highest intensity can be observed at 2θ (34.218°) with miller indices of [1 1 4]. The formation of single-phase SrFe_12_O_19_ was obtained at relatively low calcination temperature of 900 °C. There were no observed peaks corresponding to some of the reagent precursors or other secondary phases and intermediate products, except for the sample prepared at pH 8 where a minute amount of hematite Fe_2_O_3_ phase was detected and all of the samples have a good crystallinity as shown in the figure. The formation of secondary Fe_2_O_3_ phase observed for sample prepared at pH 8 had reduced the purity of SrFe_12_O_19_ to 87.8%. The Fe_2_O_3_ patterns were indexed to ICSD reference code of 98-004-1067. The presence of Fe_2_O_3_ phase is due to insufficient calcination temperature for the sample prepared at pH 8 [21]. It was found that high acidity in medium solution from pH 0 to 3 favored the formation of high crystallinity SrFe_12_O_19_ phase. The increasing pH of the sol assisted the formation of negatively charged iron gels and the adsorption of positively charged Sr ions on iron gels. Consequently, more homogeneous solution was obtained, and it results in the easy formation of SrFe_12_O_19_ phase [3]_._ Even though the formation of SrFe_12_O_19_ is easier with increased pH, heterogeneous ceramic aggregates could be formed due to localized shifts in the immediate vicinity of the complex undergoing polymerization [22]. Therefore, crystalline growth might be inhibited, therefore reducing the crystallinity from pH 4 onwards. This was shown by the increase of XRD peak intensity by the improvement of the crystallinity of SrFe_12_O_19_ prepared using pH 1 to pH 3, however slowly decreased with increased pH values from 4 to 8. The formation of crystalline SrFe_12_O_19_ after being calcined at 900 °C is attributed to the higher degree of compositional homogeneity as well as the greater heat generated from the exothermic reaction of nitrates and citric acid [21].

**Fig. 3:**
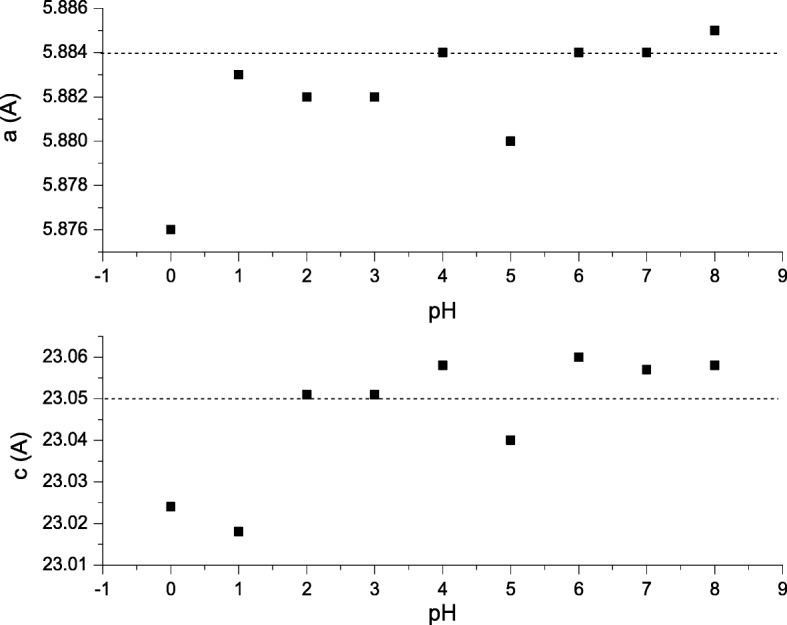
The lattice parameters *a* and *c* of SrFe_12_O_19_ nanoparticles for pH 0 to pH 8, sintered at 900 °C. The dash lines are the reference values of the lattice parameters *a* and *c*

The lattice parameter *a* and *c* value observed were not far different compared to the theoretical SrFe_12_O_19_ lattice constant where *a* = 5.8820 Å and *c* = 23.0230 Å [23] (Fig. 3). The *a* and *c* parameters observed are similar to those in Masoudpanah et al. [3] and Dang et al. [12]. The volume cell *V*_cell_ and density of XRD *ρ*_xrd_ used in this study depend on the crystallographic parameter which have a hexagonal crystal system with space group of *P63/mmc*. The *V*_cell_ were calculated using Eq. (2);

2 $$ {V}_{\mathrm{cell}}=\frac{\sqrt{3}}{2}{a}^2c $$

where *a* and *c* are the lattice constant. The theoretical density *ρ*_theory_ of sample was calculated using Eq. (3),

3 $$ {\rho}_{\mathrm{theory}}=\frac{2M}{N_AV} $$

where *M* is the molecular weight of SrFe_12_O_19_ which equals to 1061.765 g. The weight of two molecules in one unit cell is 2 × 1061.765 = 2123.53 g; *N*_A_ is the Avogadro’s number (6.022 × 10^23^ mol^−1^).

The porosity *P* of the samples can be calculated using Eq. (4);

4 $$ P=\left(\frac{1-{\rho}_{\mathrm{exp}}}{\rho_{\mathrm{theory}}}\right)\times 100\% $$

**Fig. 4:**
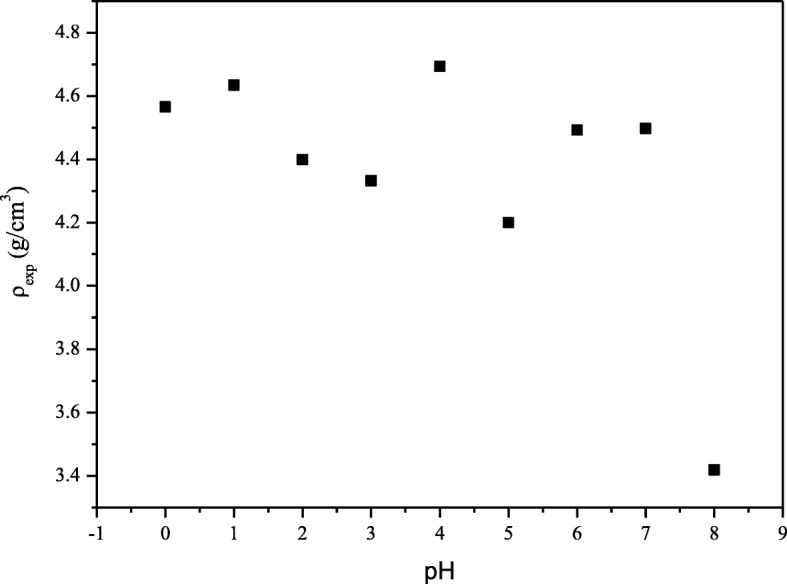
Experimental density of SrFe_12_O_19_ nanoparticles for pH 0 to pH 8, sintered at 900 °C

As the pH value increased, the experimental density of the samples *ρ*_exp_ was decreased except for some fluctuations observed for samples prepared at pH 4, 6, and 7 with optimum value of experimental density and less porosity obtained for sample prepared at pH 4. The optimum density and porosity were recorded as 4.693 g/cm^3^ and 8.15% respectively (Fig. 4, Table 1). The X-ray density shown in Table 1 is greater than the experimental density which may be due to the presence of pores created during the sintering process. The porous feature of agglomerates is also attributed to the liberation of a large amount of gas such as NH_3_ during the combustion process [24].

**Table 1:** The structural, microstructural, and magnetic parameters of the SrFe_12_O_19_ sintered at 900 °C

pH	Peak pos. 2*θ* (°)	Miller indices (hkl)	Peak width (°)	Space group	Lattice constant		*V*_cell_ (nm^3^)	*ρ*_xrd_ (gcm^−3^)	*ρ*_exp_ (gcm^−3^)	*P* (%)	*M*_s_ (emu/g)	*M*_r_ (emu/g)	*M*_s_ (emu/cm^3^)	*M*_r_ */M*_s_	*H*_c_ (Oe)	Grain size (nm)
					*a* (Å)	*c* (Å)										
0	34.32	[114]	0.19	P63/mmc	5.876	23.024	0.689	5.12	4.566	10.7	44.188	27.713	226.243	0.627	6403.6	74
1	34.20	[114]	0.13	P63/mmc	5.883	23.018	0.690	5.11	4.634	9.5	4.776	3.001	24.405	0.628	6094.7	108
2	34.21	[114]	0.13	P63/mmc	5.882	23.051	0.691	5.11	4.399	13.9	7.822	4.870	39.970	0.623	6005.8	114
3	34.22	[114]	0.16	P63/mmc	5.882	23.051	0.691	5.11	3.832	24.9	2.168	1.373	11.078	0.633	5966.1	115
4	34.20	[114]	0.16	P63/mmc	5.884	23.058	0.691	5.10	4.693	8.1	3.006	1.929	15.330	0.642	5808.6	96
5	34.25	[114]	0.16	P63/mmc	5.880	23.040	0.689	5.11	4.200	17.6	2.016	1.309	10.301	0.649	6074.8	111
6	34.18	[114]	0.16	P63/mmc	5.884	23.060	0.691	5.10	4.492	12.1	7.022	4.416	35.812	0.629	5377.0	120
7	34.18	[114]	0.18	P63/mmc	5.884	23.057	0.691	5.10	4.497	11.8	4.028	2.554	20.542	0.634	5461.2	116
8	34.17	[114]	0.18	P63/mmc	5.885	23.058	0.691	5.10	3.419	32.9	2.975	1.934	15.172	0.650	5117.7	133

**Fig. 5:**
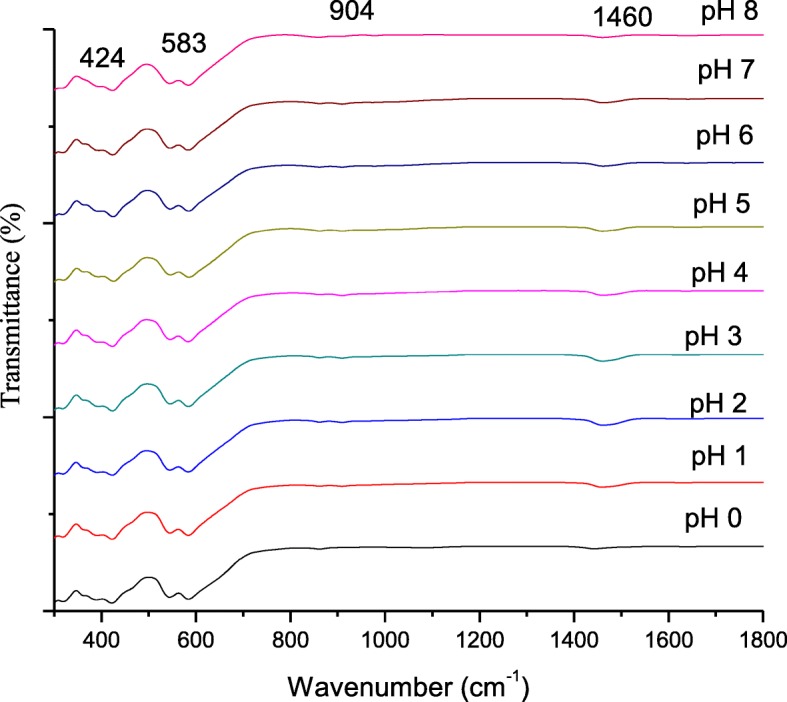
The FTIR spectra of SrFe_12_O_19_ for pH 0 to pH 8, sintered at 900 °C

The FTIR spectra of sintered SrFe_12_O_19_ at varying pH from pH 0 to pH 8 are shown in Fig. 5. The FTIR spectra of a precursor noticeably appeared in the range of 430, 583, 904, and 1446 cm^−1^ of IR characteristic bands. The absorption band at 436 cm^−1^ was indicated as a stretching band of CH_2_, proving the presence of CH saturated compound [25]. Bands at 583 cm^‑1^ show the characteristic metal oxygen vibration Sr–O Fe–O [20]. The absorption bands of range 443–600 cm^−1^ were ascribed to the formation of strontium ferrite as stretching vibration of metal–oxygen bond [26–29]. This confirms that, the SrFe_12_O_19_ was formed at a sintering temperature of 900 °C. The relatively strong and broad bands at peaks 904 cm^−1^ revealed that there was an amine functional group for N–H vibration due to the decomposition of NH_3_. Meanwhile, Pereira et al. [29] also stated that a broad vibration of Sr–O stretching indicate the formation of strontium nanoferrite. The absorption band at 1446 cm^−1^ indicates the vibrating bands of Fe–O–Fe bands due to the decomposition of metal with oxides band [25].

### Microstructural Analysis

**Fig. 6:**
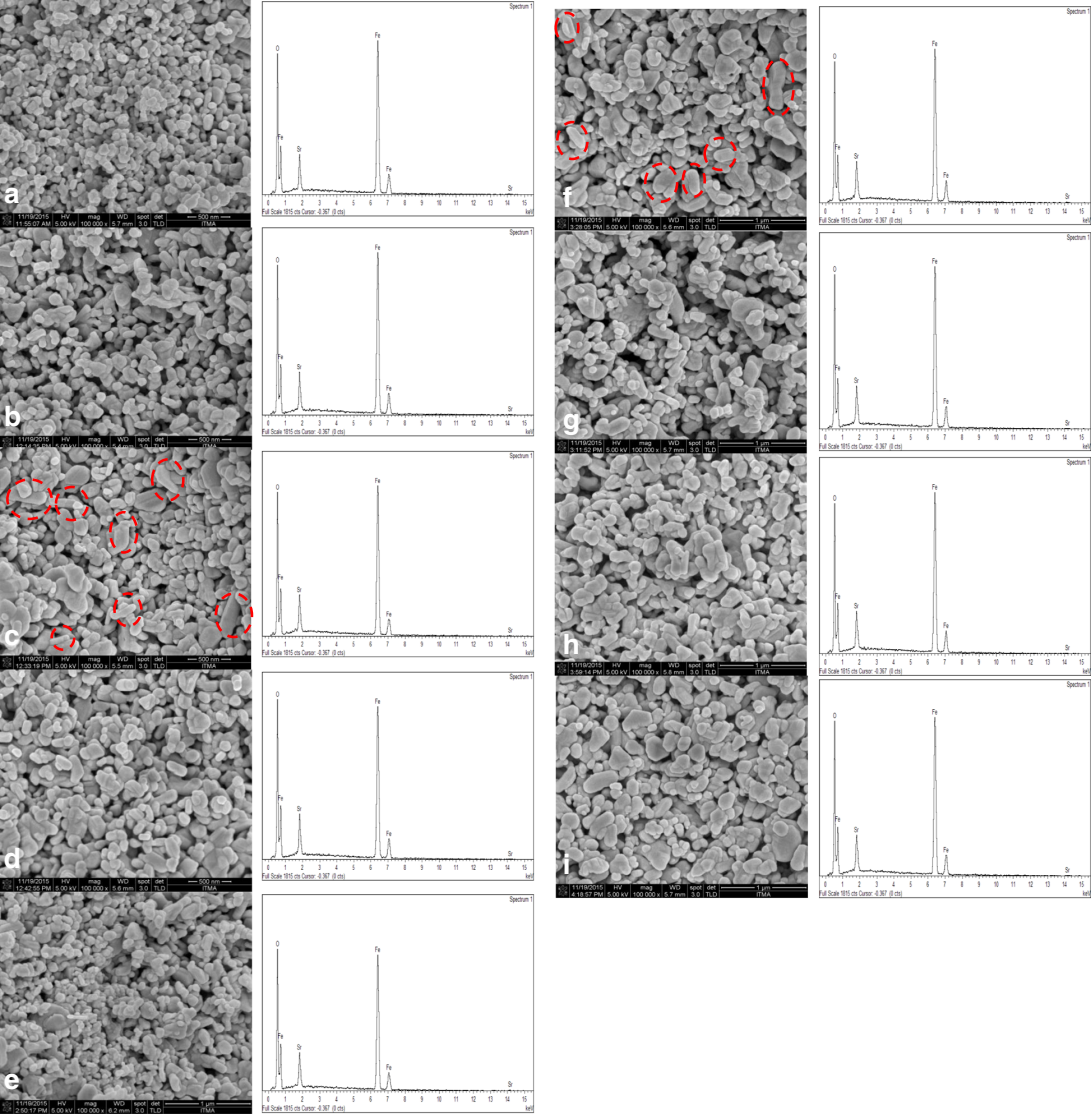
The FESEM micrographs of samples sintered at 900 °C by varying pH: **a** pH 0, **b** pH 1, **c** pH 2, **d** pH 3, **e** pH 4, **f** pH 5, **g** pH 6, **h** pH 7, and **i** pH 8

**Fig. 7:**
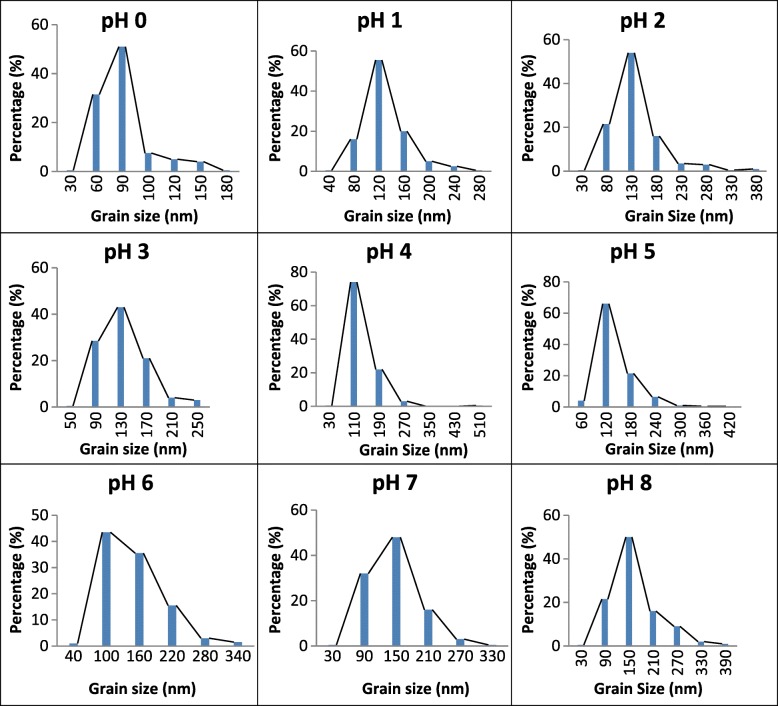
Grain size distribution for SrFe_12_O_19_ calcined at 900 °C by varying pH: **a** pH 0, **b** pH 1, **c** pH 2, **d** pH 3, **e** pH 4, **f** pH 5, **g** pH 6, **h** pH 7, and **i** pH 8

The microstructure images of bulk SrFe_12_O_19_ and the EDX spectra are shown in Fig. 6, while the grain size distributions of samples are shown in Fig. 7. The average grain sizes were found in the range of 73.6 to 133.3 nm. The average grain size of the samples does not show large variation except for samples with pH 4 and pH 8. The grain sizes were agglomerated as increasing the pH value. A relatively small and packed grain size with an average of 73.6 nm and narrowest grain size distribution among all was observed for pH 0. The grain size increased with increased pH values from pH 0 to pH 3, decreased at pH 4, and further increased until pH 8. The results are in agreement with the XRD spectra as shown in Fig. 2 that the degree of crystallinity reduced for sample at pH 4. From Fig. 6e, for sample prepared at pH 4, it shows that the grains are not homogeneously distributed and not uniformly formed.

The finest grain size exhibited highest *M*_s_, *M*_r_, and *H*_c_. The grains for samples having pH 0 were spherical in shape and in contact with another grain to form a necking structure. The contact was obvious with increase in pH values, thus showing a more elongated grain structure. The grain size/shape distributions became larger and non-uniform as the pH values increased. The histogram of the grain size distribution shifted from small grain sizes to exhibiting larger grain sizes. The increased combustion rate and heat released from reaction may also increase the crystallite size [30]. The red dotted lines in the histogram (Fig. 7) marked the average grain size of the sample. The microstructure showed some of the samples exhibited large porosity due to the presence of polyvinyl alcohol during preparation of bulk SrFe_12_O_19_ nanoferrite in pellet form as well as the liberation of gas during sample preparation.

### Magnetic Behaviors

**Fig. 8:**
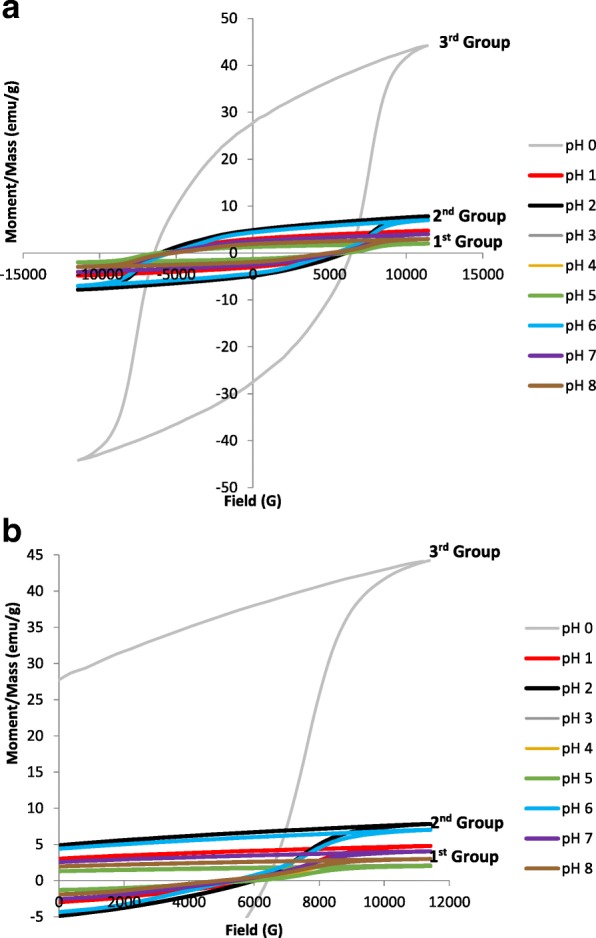
The *M*–*H* hysteresis loops of SrFe_12_O_19_ for **a** pH 0 to pH 8 and **b** close-up graph, varying pH sintered at 900 °C

The development of *M*–*H* hysteresis loop at various pH is illustrated in Fig. 8. A further confirmation of this evolution can be seen from the variation of saturation magnetization, *M*_s_, remanence, *M*_r_, squareness ratio, *M*_r_*/M*_s_, and coercivity, *H*_c_, as a function of pH tabulated in Table 1. Magnetization per unit mass is not directly related to the microstructure of the sample; therefore, magnetization per unit volume has been calculated by multiplying the magnetization per unit mass with the experimental density, *ρ*_exp_. The *M*_s_, *M*_r_, and *H*_c_ are found to be generally decreased with increasing pH by addition of ammonia in a sol–gel precursor. The decrement of magnetic parameters as pH increases could be due to the existence of large amount of diamagnetic phases of ammonia NH_3_. It seems that the main effect of the diamagnetic NH_3_ are to isolate Sr-ferrite nanoparticles from each other, thus reducing exchange interaction between them and are known to have a detrimental effect on *M*_s_ and *M*_r_. As seen previously in the “Microstructural analysis” section, the microstructure of SrFe_12_O_19_ was affected by increasing the pH value. This is in agreement with the findings reported by Yang et al. [31], where the particles became larger [32] with the increase of pH from 5 to 11. The larger particles were highly affected by strong magnetic interaction between magnetic atoms of Fe in the grains [33].

The *M*–*H* hysteresis loops in Fig. 8 have been scrutinized, and three significant groups of hysteresis loops characterized by the shapes and values of differentiated group could be observed. The first group consisted majority of the prepared samples which are samples prepared using pH 1, 3, 4, 5, 7, and 8. This group corresponded to the weak ferromagnetic properties with low values of *M*_s_ and *M*_r_*.* It is known that *M*_s_ is particularly depending on the crystallinity of the sample. This could be seen in the samples prepared using pH 4, 5, 7, and 8, where the crystallinity was reduced for the samples, thus displaying lower values of *M*_s_. Furthermore, the presence of 28.2% α-Fe_2_O_3_ impurity as a secondary phase was detected in sample prepared using pH 8, reducing the crystallinity of the sample and consequently reducing the *M*_*s*_ value. Even though the observed XRD spectra in Fig. 2 displayed high degree of crystallinity for samples prepared using pH 3, the resulting low magnetic property values might be subjected to decrease in density (see Table 1) due to the presence of pores, thus affecting the coercivity in the sample. Since *M*_s_ is related to *H*_c_ as shown in Eq. (5) [34], the *M*_*s*_ decreased when the *H*_*c*_ increased.

5 $$ {H}_c=\frac{2{K}_1}{M_s} $$

It is also known that porosity affects the magnetization process because pores work as a generator of demagnetizing field [35].

It is noticeable that pH 2 and pH 6 fell in the second group in which the samples have moderate hysteresis parameters (Fig. 8). The samples in this group exhibited similar shape of hysteresis loop with the first group but with slightly higher values of *M*_s_ and *M*_r_*.* The *H*_*c*_ values recorded for the samples prepared using pH 2 and pH 6 were 6005.8 and 5377.0 Oe respectively. The *M*_s_ values for pH 2 and pH 6 were observed as 7.8 emu/g (226.2 emu/cm^3^) and 7.0 emu/g (35.8 emu/cm^3^), respectively, whereas the *M*_r_ values for pH 2 and pH 6 were given as 4.9 emu/g and 4.4 emu/g respectively. Even though larger grain size was present in the samples, the recorded values were still low since the presence of elongated grains was detected (see the red dotted circles in Fig. 6c, g) in the samples prepared using pH 2 and 6. Since it is known that total anisotropy energy barrier depends on volume and surface anisotropy energy densities, so for a given volume of a particle, the surface area is more for elongated shape particles. Hence, the major contribution from surface to the effective anisotropy and an increase in *H*_c_ is also expected in elongated particles [36], thus reducing the *M*_s_.

The third group was detected in an only sample prepared using pH 0. Significant gap was observed between the second and the third groups, indicating the changing properties in samples within this group particularly in the *M*_s_ values. Hysteresis loop for pH 0 has the largest *M*_s_, *M*_r_, and *H*_c_ with the significant values of 44.19 emu/g (226.2 emu/cm^3^), 27.59 emu/g, and 6403.6 Oe respectively. Generally, the *M*_*s*_ values for SrFe_12_O_19_ could be ranged from 74 to 92 emu/g which are often measured in a single crystal form [8]. The value of *M*_*s*_ for sample prepared using pH 0 was relatively lower than the given values and also with previously reported studies which were 56 emu/g [37] and 53 emu/g [38], both synthesis via the sol–gel method. It is expected that the value of *M*_*s*_ in this study would be increased with further increment of sintering temperatures. However, the *H*_*c*_ value showed a relatively higher value than previous studies which were 5000 Oe [37] and 5200 Oe [38], and according to Pullar [8], no precise value is given for *H*_*c*_ as it varies too much with processing methods and grain size. Meanwhile, no significant difference of *M*_*r*_ was seen as has been previously reported which was 30 emu/g [38]. An obvious erect, larger, and well-defined hysteresis loops could be observed. It is due to the strong ferromagnetic behavior, resulting from the formation of high volume fraction of the complete crystalline SrFe_12_O_19_ phase as seen in Fig. 2. Thus, a strong interaction of magnetic moments within domains occurred due to exchange forces. This observed phenomenon can be considered as an ordered magnetism in the sample. In fact, in order to obtain an ordered magnetism and a well-formed *M*–*H* hysteresis loop, there must exist a significant domain formation, a sufficiently strong anisotropy field, *H*_a_, and optional addition contributions which come from defects such as grain boundaries and pores [39]. It is interesting to note that the broad loops in this group means substantial magnetic storage; thus, the samples possess characteristics which may be useful for practical applications [40].

**Fig. 9:**
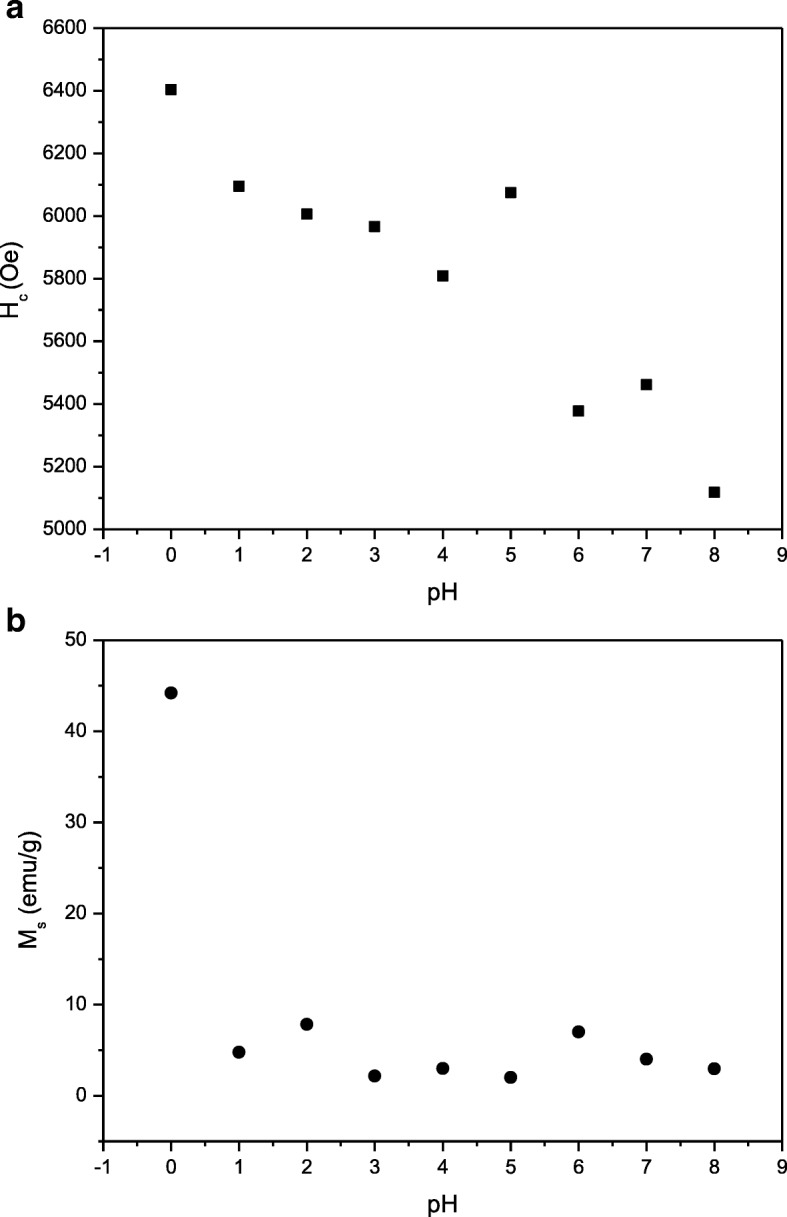
**a** *H*_c_ and **b** *M*_s_ of SrFe_12_O_19_ at varied pH sintered at 900 °C

**Fig. 10:**
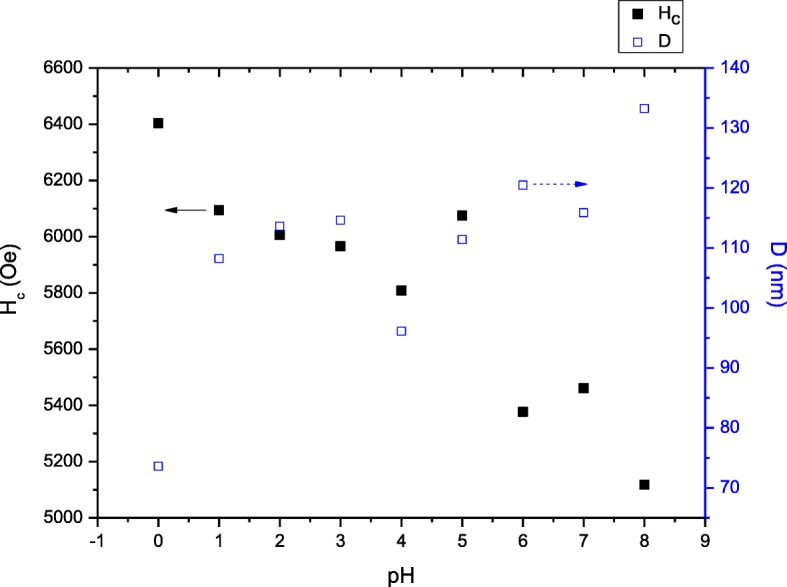
Relation of *H*_c_ and grain size of SrFe_12_O_19_ at varied pH sintered at 900 °C

The *H*_c_ variation in Figs. 9a and 10 should deserve some mention: The *H*_c_ is observed to generally reduce as pH increased. The decrease in *H*_c_ with increasing pH can be attributed to decrement of magnetocrystalline anisotropy with anisotropic Fe^2+^ ions locating on a 2a site and the enlargement of the grain size and is evident in FESEM micrographs (Fig. 6). Furthermore, at pH 8, the coercivity *H*_*c*_ which is 5117.7 Oe was recorded due to the presence of 28.2% α-Fe_2_O_3_ impurity (Fig. 2). The decrease in *H*_c_ was due to the presence of impurity α-Fe_2_O_3_ which affected the crystalline and grain boundary since it has been reported that the *H*_c_ could be affected by important parameters such as particle size, ion substitution, morphology, interface structure, crystal defects, magnetocrystalline anisotropy, and strain [41]. The squareness ratio, *M*_r_*/M*_s_, is calculated from the magnetic data and tabulated in Table 1. Generally, a large *M*_r_*/M*_s_ value is preferred in many applications such as magnetic recording media of high density and permanent magnet [42]. The calculated *M*_r_*/M*_s_ in this study was found to be in the range of 0.63 to 0.65, indicating that all the samples are predominantly in single magnetic domain structure [43]. *M*_r_*/M*_s_ equal to or above 0.5 indicates that the particles are in the single magnetic domain and below 0.5 may be attributed to the formation of multidomain structure [43, 44].

## Conclusions

Single-phase nanoparticles of SrFe_2_O_19_ prepared using different pH were successfully synthesized by sol–gel method. The effects of structural, microstructural, and magnetic behavior of SrFe_2_O_19_ were studied by modifying the pH values at the fix sintering temperature of 900 °C. From this study, it can be concluded that pH values play an important role in the formation of single-phase SrFe_12_O_19_ which required pH not more than 7 and, by increasing pH from 0 to 3, the formation of SrFe_12_O_19_ is favored. SEM micrographs exhibited a circular crystal type of SrFe_2_O_19_ with average grain size in the range of 73 to 133 nm. The single-phase SrFe_2_O_19_ with optimum magnetic properties are observed in sample prepared at pH 0 which displayed best in-plane saturation magnetization of 44.188 emu/g and remnant magnetization of 27.593 emu/g and with high coercivity of 6403.6 Oe.

## Abbreviations

*ρ*_exp_: Measured sample’s density
*ρ*_theory_: Theoretical density
*ρ*_*w*_: Density of water
*ρ*_xrd_: Density of XRD
*a*: Lattice parameter
C: Carbon
*c*: Lattice parameter
C/N: Citrate to nitrate
C_3_H_4_(OH)(COOH)_3_: Citric acid
EDX: Energy-dispersive X-ray
Fe: Iron
Fe(NO_3_)_3_: Iron(III) nitrate
Fe_2_O_3_: Hematite
FESEM: Field emission scanning microscope
FTIR: Fourier transform infrared
H: Hydrogen
*H*_a_: Anisotropy field
H_c_: Coercivity
IR: Infrared
*K*_*1*_: Anisotropy constant
*M*: Molecular weight
*M*_r_: Remanence
*M*_r_*/M*_s_: Squareness ratio
M_s_: Saturation magnetization
N: Nitrogen
*N*_A_: Avogadro’s number
NH_3_: Ammonia
NH_4_OH: Ammonia
O: Oxygen
*P*: Porosity
Sr: Strontium
Sr(NO_3_)_2_: Strontium nitrate anhydrous granular
SrFe_12_O_19_: Strontium ferrite
*V*_cell_: Volume cell
VSM: Vibrating sample magnetometer
*W*_air_: Sample’s weight in air
*W*_water_: Sample’s weight in water
XRD: X-ray diffraction
